# Notes on Preparations for the Sick

**Published:** 1890-09

**Authors:** 


### Notes on Preparations for the Sick.

**Exalgine.**—B. Kühn, London.

This new analgesic has now established itself in the list of remedies of which morphia is the chief and indispensable but not always admissible.

Since Professor Fraser published his favourable results from the action of very small doses of half a grain and upwards, the drug has come into demand, but the results are not always satisfactory. Nothing is more difficult than to estimate the value of any drug in relieving minor degrees of pain not severe enough to require a more powerful anodyne like morphia. We suspect that many of the marvellous results brought about by doses of half a grain to two grains of exalgine have been due to other than the anodyne effects of the drug; viz., "the fallacies in estimating therapeutic effects when reliance has almost solely to be placed on the statements of patients."

In an article on Exalgine in the *Medical Press and Circular* (February 26th, 1890), Dr. Alfred S. Gubb points out that the only precautions to be observed in prescribing the drug are the following:

1. To commence with doses not exceeding from two to five grains (or even less).
2. Never to give more than from ten to twelve grains in the twenty-four hours.
3. To consider the febrile condition as a formal contra-indication.

It can be given as a powder in wafers or "cachets." Though but sparingly soluble in cold water, it is readily dissolved in the stomach, owing to the acidity of the gastric juice, a fact ascertained experimentally by M. Gaudineau. The most convenient form, however, in which to order exalgine is as a mixture, the drug to be dissolved in a small quantity of spirit before adding the water.

The formulæ most employed are the following:

℞ Exalgine, gr. xij;
Spirit of peppermint, q.s.;
Syrup of orange flowers, ℥j;
Cinnamon water to ℥vj.

℞ Exalgine, gr. xij ;
Rum or whisky, ℥ss ;
Dissolve and then add—
Simple syrup to ℥j ;
Distilled water, ℥vj.

℞ Exalgine, gr. xij ;

Tincture of orange-peel, ʒij ;

Syrup, ℥ss ;

Distilled water to ℥vj.

A tablespoonful of either of these mixtures thus contains one grain of exalgine.

Our own experience of this drug would not lead us to rely on it when immediate steps for the relief of severe pain are required. It is, doubtless, a useful drug, and may frequently enable the patient to dispense with the more active analgesics. An addition to our arms for the combating of pain is always welcome; and this new one will have a range of utility, although that range seems likely to be limited to the minor degrees of evanescent pains for which something must be done, but which are not severe enough to require the more trustworthy but objectionable alkaloids of opium.

---

**The Standard Malt Extract.**—The Standard Malt Extract Company, London.

This preparation is said to be made exclusively from the finest English malt is guaranteed to be absolutely pure, and to be, in diastatic power, at least three to four times the strength of any other extract. As the presence of Diastase constitutes the chief value of all malt extracts, and analysis shows that the preparation contains an exceedingly large amount of the amylolitic ferment, we cannot doubt that its worth as a digestive of starchy foods is great. The maltose and soluble phosphates give it also an appreciable nutritive power in addition to its value as a digestive of other foods.

The combination with cod-liver oil is well known to be of the greatest value in consumption and other wasting diseases.

---

**Cocoa Essence.**—Cadbury Brothers, Birmingham.

In hardly any respect has there been greater progress of late years than in the better quality and increased purity of food. Accum's celebrated treatise, *Death in the Pot*, was once rigidly true; but not in our day. Everything which the unhappy Englishman bought was liable to adulteration: his clothes were not what they professed to be; his food was falsified to such a degree, that whatever else it might be, it certainly never was what it purported to be, and what he had a perfect right to expect it was; his sugar was rich in sand; his coffee was mixed with clay imitations; his tea might never have known any other clime than that of his native land; as for his spices, the chemist—most unscrupulous and ingenious of men—had more to do with their preparation than the consumer would have cared to hear. As for actual figures, a distinguished medical author in the *Quarterly Review* once stated, and supported his assertion with apparently incontestable facts, that 750,000 pounds of impure tea were sold in England every year. Were not the subject horrible and revolting, it would be possible to bring forward such an array of adulterations that one might well ask what analysts and medical officers of health were doing, and what could possess the public to submit to such scandalous imposition? We hope that matters are somewhat better now, but we are anything but convinced; for whenever an inquiry is made, the results are very far from satisfactory, and it is still the exception, not the rule as it ought to be, to find that the goods offered by even the best firms are what they should be. We do not regard all adulterations as equally heinous—no such thing. The substitution of flour for a more expensive and valuable article is a fraud, true, and should be severely punished; but it is not injurious. When, however, it comes to potent chemicals being systematically added, what words can sufficiently convey our indignation? We have of late seen enough to make us suspect that much still goes on which would not bear the light; and that a book, remarkable for the deep insight it would give into human villainy on the one hand, and human long-suffering and stupidity on the other, could be put together.

Medical practitioners are often appealed to for an opinion by their clients; and it would be well indeed were they to study the present subject a little more carefully, and to set their faces resolutely against all adulterations, no matter whether that adulteration only consisted of the substitution of cheaper for dearer articles, or went the length of subjecting the consumer to a course of severe medical treatment of a kind for which he had no need.

Bad as were the adulterations of tea, coffee, sugar, spices, and flour, it was found that cocoa, which is now coming into such general use, was even less often to be got pure than most other articles. These adulterations mainly consisted of sugar, starch, and flour; red colouring matters were not infrequent, while ferruginous earths were also commonly found; although brick-dust—which we believe Dr. Parkes thought was used, at least his words will bear that construction—never was mixed with cocoa, at least so we are assured by eminent manufacturers.

Cocoa of the most excellent quality and of absolute purity is now to be bought at very moderate prices, and no purchaser need be at a loss to get an article to which the severest tests can be applied and which will come out triumphantly from the ordeal.

Nevertheless, we were startled not long since at receiving a pamphlet, bearing on its front page the names of some distinguished chemists, and addressed to the *medical profession alone*, vaunting some well-known foreign-manufactured cocoas, which were distinctly stated to be freely adulterated and to contain a considerable addition of alkaline salts. Surely medical readers do not need to be reminded that soda and potash in free doses cannot be taken with impunity day after day. The healthy body does not require daily doses of drugs—valuable, no doubt, in real emergencies, but injurious when indulged in at other times. But we are not so much concerned with the actual injury likely to be done by the adulteration, as with the principle involved. The recent great improvements in the preparation of cocoa by removing the superfluous oil has so much increased the solubility of this nutritious beverage, that the last excuse for the addition of alkalies and starch is gone; and the presence of these salts, besides being possibly deleterious to some constitutions, cannot answer any purpose whatsoever, except to give an appearance of fictitious strength to the resulting infusion or decoction.

Our contention in short is, that anything which, like coffee, tea, or cocoa, purports to be absolutely pure, should always be offered to the general public unadulterated. Whatever we may think of mixtures of coffee or cocoa, we do not feel called upon to denounce them as long as it is clearly understood that they are mixtures, and do not profess to be pure, though the public would obviously gain were it in all cases distinctly stated of what the mixture consists. We trust that the profession will recognise its duty, and do its utmost to discountenance the use of all adulterated articles whatever defence of medication may be offered. Cocoa should be perfectly pure, like sugar, spices, bread, and a hundred other foods; and any encouragement of more or less medicated and adulterated foods and drinks, though it may not be a crime, is certainly a sin. We are determined to contend in all legitimate ways for absolute purity; we shall at any rate fight against all additions not imperatively demanded by the exigencies of manufacture. Our remarks must not, however, be supposed to apply to chocolate-creams and other preparations of manufactured cocoa, as long as they only consist of wholesome sugar and flavouring ingredients, and are understood to be mixtures.

Cadbury's **Cocoa Essence** is described as a genuine cocoa about three times the strength of the best homœopathic or prepared cocoas, in which the mass of fatty matter, comprising fifty per cent. of the cocoa-nib, is disguised by the addition of arrowroot, sugar, &c.

Why any cocoa should be called homœopathic, we are at a loss to understand, and it is much to be desired that so meaningless or misleading a name should be discarded. Perhaps the late Andrew Wynter, whose horror of adulteration was one of the noblest traits in his fine character, did not exaggerate when he wrote that, "as far as we can see, what is called homœopathic cocoa is only distinguished from other kinds by the small quantity of that substance contained in it."

The cocoa essence makes an agreeable beverage, refreshing and nutritious; and is easily prepared by the addition of either boiling water or milk. It has recently been strongly recommended by Dr. William Faussett as of use for the feeding of infants, being cheap, simple, and readily available. He states, "that as cocoa contains all the elements indispensable for the growth and development of the body, and can always be presented in a fluid form, it is, next to milk, preferable to all other natural substances as an article for infant aliment."

